# Microbiota profile in organs of the horseflies (Diptera: Tabanidae) in Northeastern China

**DOI:** 10.3389/fmicb.2024.1467875

**Published:** 2024-09-24

**Authors:** Hong-Yu Qiu, Qing-Bo Lv, Chun-Ren Wang, Hao Ju, Chun-Feng Luo, Shun-Shuai Liu, Mu-Han Na, Qiao-Cheng Chang, Jia-Fu Jiang

**Affiliations:** ^1^School of Public Health, Shantou University, Shantou, China; ^2^Key Laboratory of Bovine Disease Control in Northeast China, Ministry of Agriculture and Rural Affairs, College of Animal Science and Veterinary Medicine, Heilongjiang Bayi Agricultural University, Daqing, China; ^3^State Key Laboratory for Diagnosis and Treatment of Severe Zoonotic Infectious Diseases, Key Laboratory for Zoonosis Research of the Ministry of Education, Institute of Zoonosis, and College of Veterinary Medicine, Jilin University, Changchun, China; ^4^State Key Laboratory of Pathogen and Biosecurity, Beijing Institute of Microbiology and Epidemiology, Beijing, China

**Keywords:** arthropods, Malpighian tubules, microbiota, midgut, ovaries, tabanids

## Abstract

Tabanids, commonly known as horseflies and belonging to the family Tabanidae, are blood-feeding arthropods (BFA) found worldwide. They are known for their ability to mechanically and biologically transmit various animal pathogens. Tabanids are potential vectors for diseases such as *Francisella tularensis, Anaplasma marginale, Theileria* spp., and contributors to lumpy skin diseases. Despite their involvement in common BFA studies, tabanids have not been extensively explored in microbiome research. In this study, the microbiota structure and composition in various organs of four distinct genera of tabanids: *Atylotus*, *Haematopota*, *Tabanus*, and *Hybomitra* were examined. High-throughput sequencing of the bacterial 16S rRNA gene was performed to gain insights into the microbial communities associated with the different tabanid species. Result display that microbiota composition and diversity, including Firmicutes, Proteobacteria, and Bacteroidetes, varied significantly among the different organs, with the ovaries exhibiting significantly higher diversity. Apart from the *Haematopota* genus, *Tenericutes* were enriched in the midgut of other tabanid species, whereas the Malpighian tubules exhibited a higher abundance of Bacteroides. Notably, the ovarian microbiota structure was conserved among the four tabanid species, indicating its potential association with reproductive development. Evaluation of the potential pathogen risk revealed putative pathogens in over 100 genera associated with these tabanid commensal organisms. Twenty genera were annotated as zoonotic agents with a high abundance of *Citrobacter* and *Brucella*, highlighting the presence of this important group of zoonotic pathogens. Functional predictions of vector-microbiota interactions indicate that microbiota significantly affects vector biological traits and can influence pathogen transmission via direct interactions or by regulating host immunity and nutrition. For the first time, the distribution characteristics and functions of four genera of horsefly microbiota were analyzed, revealing the presence of multiple potential pathogenic microorganisms. These findings provide valuable insights for future research and the development of symbiotic-based strategies to control insect-borne diseases among tabanids.

## 1 Introduction

Tabanids, commonly known as horseflies, belong to the family Tabanidae and are widespread blood-feeding arthropods (BFA) within the order Diptera, encompassing four subfamilies and 144 genera (Sevidzem et al., 2021). They are crucial in transmitting pathogens through mechanical and biological means, affecting wild animals, domestic livestock, and humans (Maity et al., 2017; Whyte et al., 2020). Members of the family Tabanidae are vectors implicated in transmitting more than 80 viral, bacterial, and protozoan agents (Krinsky, 1976), rendering them of notable medical and veterinary significance because of the substantial risks they pose to livestock and wildlife.

Numerous metagenomic and 16S-rRNA amplicon sequencing studies have extensively explored the symbiotic microbiota relationships in various arthropods (Shi et al., 2022; Wang et al., 2023; Song et al., 2022; Ahmad et al., 2022; Murakami et al., 2018). The microbiota functionally complements the host biology and helps synthesize essential vitamins and cofactors (Song et al., 2022). The focus has predominantly been on well-known species such as bees, mosquitoes, and ticks (Feng et al., 2022; Liberti et al., 2022; Kurokawa et al., 2020). These species tend to receive more attention because they transmit diseases such as *Plasmodium* spp. and other deadly pathogens, making them economically and medically important (Wang et al., 2023). In contrast, hematophagous arthropods such as horseflies, deerflies, and blackflies have received comparatively less attention. Despite being less harmful than well-known vector insects, these arthropods remain crucial for pathogen transmission, particularly in endemic regions with inadequate medical and sanitary conditions (Rodrigues et al., 2022; Olkeba et al., 2022; Sitarz et al., 2022). The application of high-throughput sequencing technology provides a promising avenue for investigating the microbial diversity of arthropods (Gómez et al., 2022; Swe et al., 2019; Estrada-Peña et al., 2018). Gaining deeper insight into the intricate relationship between arthropods and their microbiota will contribute to a broader understanding of the ecology and evolution of these organisms.

Tabanids exhibit remarkable diversity, comprising over 4,500 species and subspecies, and are widely distributed worldwide. *Tabanus* has a global distribution and is particularly abundant in subtropical and tropical regions (Mullens et al., 2022; Keita et al., 2020; Votýpka et al., 2019). *Haematopota* spp. and *Chrysops* spp. are the most prevalent in Europe (Dörge et al., 2020) whereas in Northeast China, the dominant species is *Hybomitra* sp., especially in the Palearctic region, followed by *Tabanus*, *Haematopota turkestanica*, *Haematopota* and *Atylotus*. Tabanids are 6–30 mm long and possess wings and large compound eyes. They are primarily active around animal colonies during the daytime in the summer. Many female tabanids require blood for oogenesis. Upon biting, the saliva containing various anticoagulants and allergens are injected into the host (Veraldi and Esposito, 2017; Maity et al., 2017), leading to allergic reactions and pain. Consequently, they pose significant challenges to grazing areas, causing weight loss, discomfort, and agitation. Moreover, tabanids have medical significance as vectors of numerous viruses, bacteria, parasites, and pathogens (Keita et al., 2020; Baldacchino et al., 2014) contributing to the spread of diseases in humans and animals through biological and mechanical transmission. Studies have highlighted their role in transmitting Francisella tullarensis, *Anaplasma marginale Theileria* spp., and causing lumpy skin disease (Janse et al., 2017; Hornok, 2008; Sohier et al., 2019).

The microbiota plays a crucial role in the development of BFA and can significantly affect their vectorial capacity and host immune system signaling (Husnik, 2018; Sonenshine and Stewart, 2021). They colonize various tissues within insects. For instance, in mosquitoes, the salivary glands exhibit the highest microbiota diversity (Sharma et al., 2014), a feature closely linked to their life history traits. The microbiota present in the midgut or salivary glands can interact directly with pathogens, thereby affecting the vectorial capacity of BFA (Gómez et al., 2022; Aguilar-Díaz et al., 2021). Consequently, manipulating the bacterial populations inhabiting these BFAs has emerged as a promising strategy for developing novel strategies to control the transmission of vector-borne diseases.

Despite the numerous microbiome studies conducted on common BFA, similar investigations in tabanids remain undocumented. Given the potential effect of bacteria on vector capacity, understanding the composition of the microbiota within tabanids could aid in assessing whether certain tabanid populations are more prone to transmitting pathogens than others (Zhu et al., 2024). In this study, we assessed the microbiota structure of four tabanid species by analyzing their presence in various organs across the four tabanid genera. These findings contribute to advancing our understanding of the bacterial communities associated with tabanids, thereby establishing a foundation for future research and implementing control measures targeting tabanids.

## 2 Methodology

### 2.1 Tabanids sample collection and cleaning

Tabanids were captured near horse and cattle farms in Daqing City, Heilongjiang Province, Northeastern China, using the net method from July to September 2021. Collectors held the end of the net handle and waved it in an “X” pattern, allowing for easy maneuvering while walking. Tabanids of the same genus were placed in specialized cages to shield them from direct sunlight and ensure their activity. Species identification was based on morphological characteristics, including the shape of the basal scapula of the head, as well as the length, width, and degree of dorsal protrusion of the antennae. We also examined the colour of the back plate on the abdomen and wing pattern (China fauna editorial committee of the Chinese Academy of Sciences, 1998). The cleaning procedures were as follows: (1) The tabanids were placed into a sterile centrifuge tube, followed by the addition of 75% ethanol solution. The tube was then gently shaken for 1 min; (2) the tabanid samples were transferred to a new centrifuge tube, and 0.1% sodium hypochlorite solution was added. The tube was again shaken gently for 1 min; (3) the samples were transferred once more to a new centrifuge tube and cleaned twice with distilled water; (4) distilled water was added again, and after gentle agitation, the liquid was poured out for preservation; and (5) and the cleaned tabanids were fixed onto wax plates and dissected under sterile conditions, and tissue samples were collected from the Malpighian tubules, midgut, and ovary. The tissue samples were stored at −80°C for subsequent use. A total of 432 tabanids were collected. Among them, 135 tabanids were used in the study, including 40 *T. griseinus*, 45 *Atylotus* sp., 25.

*H. turkestanica* and 25 *Hybomitra* sp. After dissecting the tabanids five samples was combined into one pool and submitted to sequencing. All tabanids were adults, and the detailed sample information is shown in Table 1.

**Table 1:** The information of the tabanids collected in this study.

Pools ID	Number	Species	Sex	Organ
T1–T8	8	*Tabanus griseinus*	Female	Malpighian tubules
T9–T16	8	*Tabanus griseinus*	Female	Midgut
T17–T24	8	*Tabanus griseinus*	Female	Ovary
A1–A9	9	*Atylotus* sp.	Female	Malpighian tubules
A10–A18	9	*Atylotus* sp.	Female	Midgut
A19–A27	9	*Atylotus* sp.	Female	Ovary
HA1–HA5	5	*Haematopota turkestanica*	Female	Malpighian tubules
HA6–HA10	5	*Haematopota turkestanica*	Female	Midgut
HA11–HA15	5	*Haematopota turkestanica*	Female	Ovary
HY1–HY5	5	*Hybomitra* sp.	Female	Malpighian tubules
HY6–HY10	5	*Hybomitra* sp.	Female	Midgut
HY11–HY15	5	*Hybomitra* sp.	Female	Ovary

### 2.2 DNA extraction and 16S rRNA sequencing

DNA was extracted from the Malpighian tubules, midgut, and ovary tissues using the DNA Stool Mini Kit, following the manufacturer’s protocol. Subsequently, the extracted DNA was utilized for Illumina sequencing using primers 338F: 5′- ACT CCT ACG GGA GGC AGC AG-3′ and 806R: 5′- GGA CTA CCA GGG TATC-TAA TCC-3′, targeting the 16S (V3–V4) region. PCR products were subjected to 250 bp paired-end sequencing on an Illumina NovaSeq 6,000 platform.

### 2.3 Sequence data processing and generation of amplicon sequence variants table

Raw reads were initially filtered using Trimmomatic (v0.33) (Bolger et al., 2014). Subsequently, the primer sequences were identified and eliminated using Cutadapt (v1.9.1) (Martin, 2011), resulting in the generation of high-quality reads without primer sequences. The clean reads were then spliced and clustered on the QIIME2 platform (Bolyen et al., 2019) using the DADA2 algorithm (Callahan et al., 2016) to obtain amplicon sequence variants (ASVs). Taxonomic annotation of the feature sequences was processed using a Bayes classifier with SILVA (release 138.1) as the reference database (Quast et al., 2012). The Shannon diversity index and Bray-Curtis distance of the samples were calculated using the “diversity” and “vegdist” functions in the R package: vegan. Permutational multivariate analysis of variance (PERMANOVA) was performed using the adonis function in vegan. The ggplot2 package was used to visualize data such as species composition and principal component analysis. Statistics on the composition of each sample were calculated at various taxonomic levels, including phylum, class, order, family, genus, and species. The abundance of each species in the samples and distribution histograms at each taxonomic level were obtained using QIIME2.

### 2.4 Statistical analysis

All analyses were conducted using R version 4.2.1. Rarefaction curves based on the number of feature sequences were generated using “estimate R” function in the package vegan v2.5–7 (Dixon, 2003). The profile was transformed into a table of relative abundances prior to statistical analyses. Wilcoxon rank sum test was employed to assess differences in species between groups, and a *p*-value of <0.05 was considered statistically significant. The Shannon index was used to evaluate microbiota diversity. Principal coordinate analysis (PCoA) was conducted based on the Bray-Curtis distance and permutational multivariate analysis of variance (PERMANOVA) with a permutation of 999. Linear discriminant analysis of effect size (LEfSe) was performed to identify the key species that most likely explained the differences between the groups. Multiple bacterial pathogen detection (MBPD), which can detect a broad range of animal, plant, and zoonotic pathogens based on 16S rRNA gene sequencing (Yang et al., 2023), was employed to explore putative pathogens in horsefly microbial communities.

## 3 Results

### 3.1 Morphological characteristics

**Figure 1:**
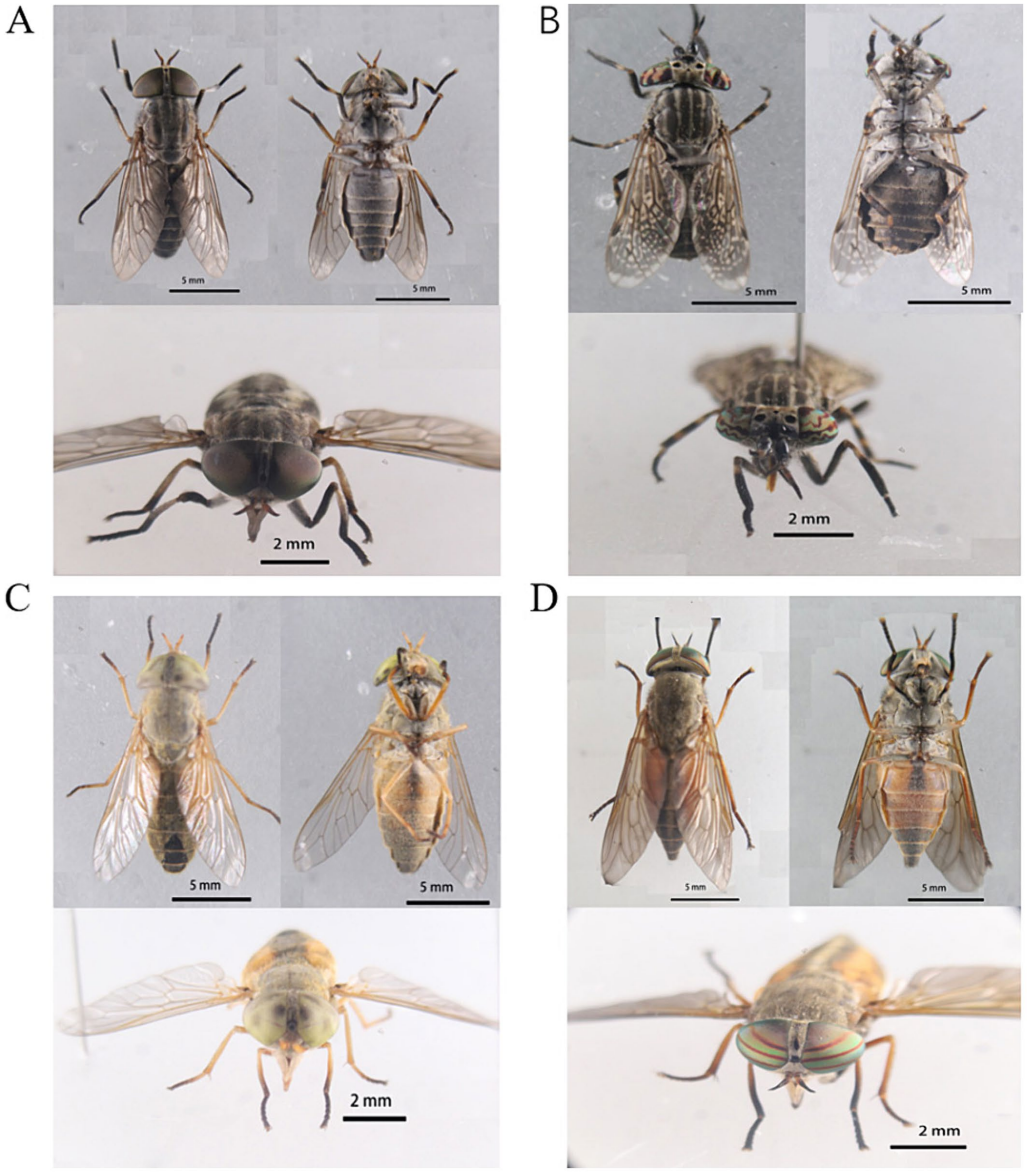
Morphological characteristics of tabanids. **(A)** *Tabanus griseinus.* **(B)** *Haematopota turkestanica.* **(C)** *Atylotus* sp. **(D)** *Hybomitra* sp.

Phenotypic sex determination relies on secondary sex characteristics, particularly eye shape. Morphological identification was conducted individually for each collected specimen, using morphological descriptions to ascertain the genus or species level. The captured tabanids were classified into four genera: *Atylotus*: Usually small sized fly, frons with spotted calli or without it. Colour of eyes in living specimen green or yellow. Basal plate of flagellum broad and obtuse dorsal angle. Basicosta pale to brown setulose. *Haematopota*: Generally small and slender flies of brownish to blackish grey in colour; eyes with several wavy bands in live condition; frons with velvety black spot on each side above the frontal callus and often a mid-frontal spot above these; picture wing pattern, i.e., dark wing with pattern of pale spots; mid tibiae and hind tibiae often with pale rings. *Tabanus*: Frons with prominent callus. *Hybomitra*: Medium sized fly; vertex with ocellar tubercle; eyes with dense pubescence and 3 green or purple band in live condition; basal and median callus usually broad; body blackish to dark greyish often with orangish side markings in at least 2 to 3 anterior abdominal segments. Two of these were identified at the genus level (*Atylotus* sp. and *Hybomitra* sp.), while the other two were identified at the species level (*Haematopota turkestanica* and *Tabanus griseinus*). The morphological characteristics of the cells are depicted in Figure 1A.

### 3.2 Summary of 16S-rRNA sequencing

After dissecting the tabanids We characterized different organs bacterial community composition by sequencing the V3–V4 region of the 16S rRNA (16S) gene. In total, 7,516,786 pairs of raw reads were obtained. After quality control, 7,486,165 clean reads were obtained, with an average of 77,981 clean reads per sample. A total of 1,338 ASVs were obtained following the splicing and clustering of clean reads. Notably, more than 1,000 ASVs were shared among the different organs of the four tabanid species, indicating the presence of a relatively stable core community within each organ. Taxonomic classification revealed that nearly all ASVs could be classified above the genus level, indicating that the majority of the tabanid microbiota comprised known bacteria (Figures 1B–D).

### 3.3 Diversity of microbiota in Tabanidae

**Figure 2:**
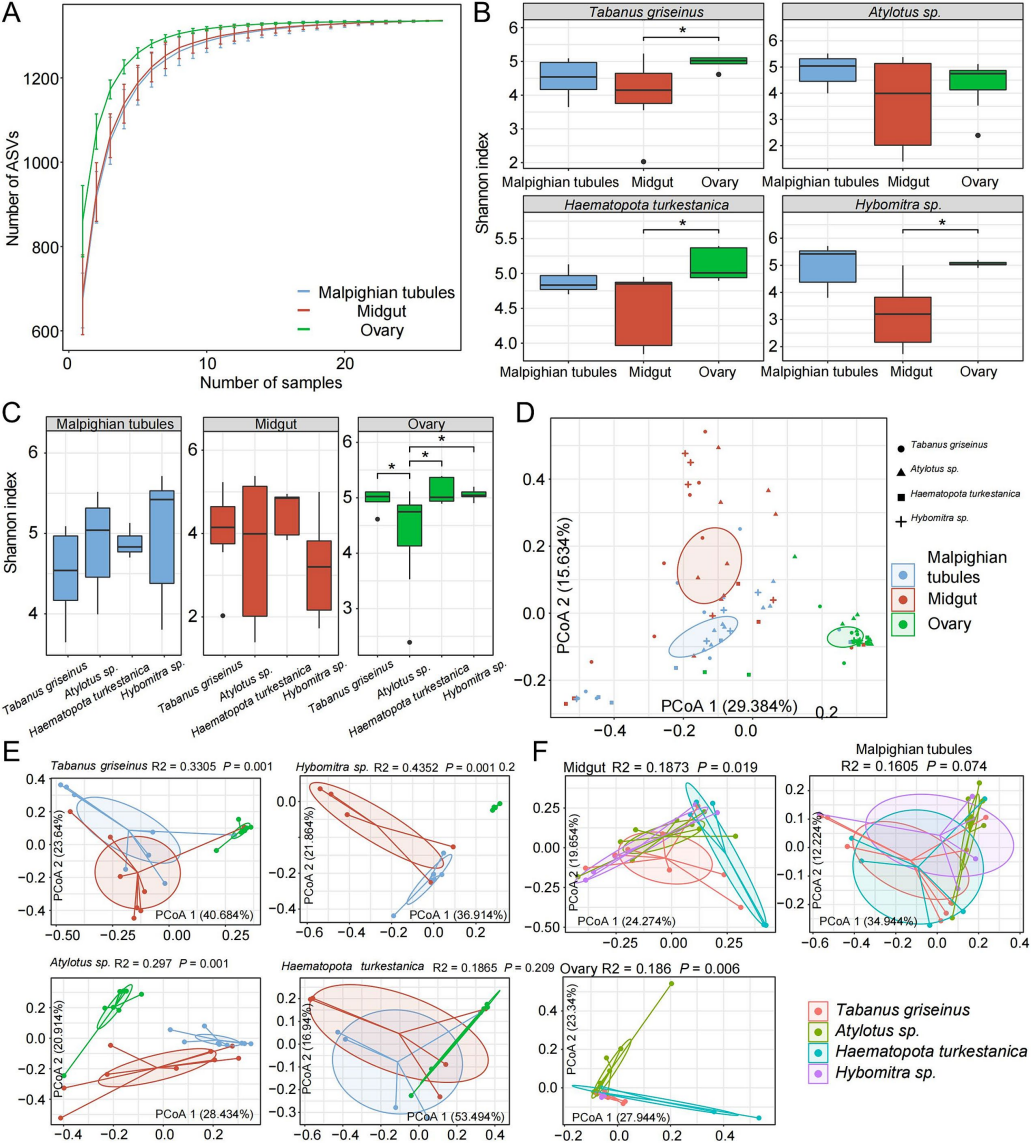
Symbiotic microbial diversity in tabanids. **(A)** The dilution curve shows that the number of observed feature sequences increases with the number of samples. **(B,C)** Show a comparison of the *α* diversity of microbes across different organs and species, respectively. **(D)** PCoA analysis based on Bray-Curtis distances revealed a distinct separation among samples, with four shapes representing different species and three colors used to distinguish various organ groups. **(E,F)** Show the separation between different organs and different species, respectively. *R*^2^ and *p* values represent the results of PERMANOVA.

To investigate the diversity of the microbiota in Tabanidae, the microbiota present in the midgut, Malpighian tubules, and ovary of four genera of Tabanidae were analyzed. The rarefaction analysis indicated that the sampling number curve for each organ tended to saturate at 20 (Figure 2A). Moreover, the number of species observed in the ovaries exceeded that in the other two organs with the same sample size, indicating higher species richness in the ovaries.

Subsequently, the Shannon index was used to evaluate the microbial diversity in each organ. The midgut exhibited significantly lower microbial diversity than the other organs, whereas the ovaries displayed the highest microbial diversity (*p* < 0.05). The rank-sum test revealed that *Tabanus griseinus*, *Hybomitra* sp., and *Haematopota turkestanica* exhibited significantly higher microbiota diversity in their ovaries than in the other organs (*p* < 0.05; Figure 2B). Furthermore, microbiota diversity within the same organ among different tabanids was compared. The microbiota diversity of *Atylotus* sp. ovaries was low (*p* < 0.05, compared with other groups), whereas the differences among the other groups were not statistically significant (Figure 2C).

PCoA was conducted using the Bray-Curtis distance metric revealed significant effects of different organs and tabanids on the microbiota composition (Figure 2D). Notably, the organs exhibited stronger explanatory power for microbiota variation (R2 = 0.2264, *p <* 0.001) than the tabanids. Further analysis of the characteristics of microbial diversity among the different tabanids involved the calculation of the distance between samples at the species and organ levels. We observed significant differences in the *β*-diversity of the microbiota across different organs within the same species (Figure 2E). Among different species, the β-diversity of the Malpighian tubules microbiota is relatively similar, whereas the midgut and ovary microbiota exhibit substantial differences (Figure 2F).

### 3.4 Composition of microbiota in Tabanidae

**Figure 3:**
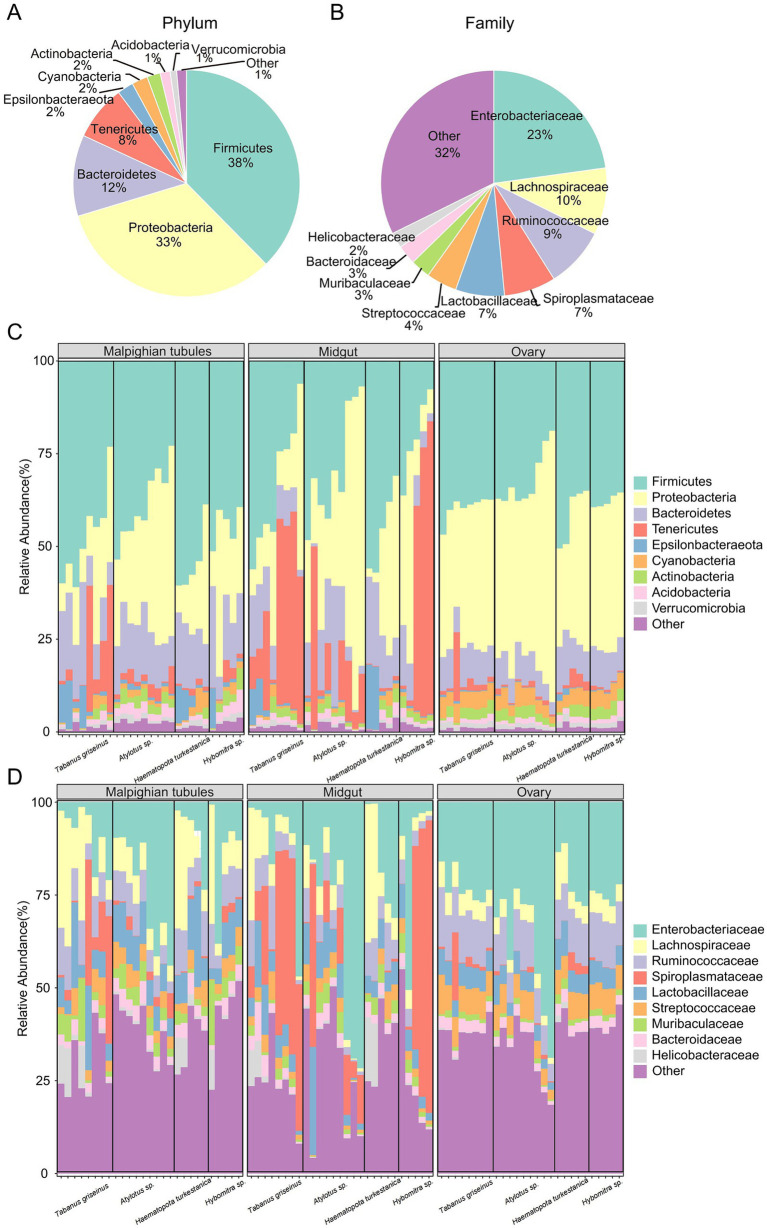
Microbiota composition in tabanids. **(A,B)** Show bacterial taxa in all samples, at phyla and family level, respectively; **(C,D)** Show bacterial taxa in difference group, at phyla and family level, respectively. Only the top ten phyla or families with the highest abundance are shown.

A total of 1,338 ASVs were classified into 20 phyla, 225 families, and 506 genera. Firmicutes (37.6% ± 13.7%), Proteobacteria (32.7% ± 20.4%) and Bacteroidetes (11.6% ± 6.3%) were the dominant phyla. Other phyla, including Tenericutes (7.8% ± 17.4%), Epsilonbacteraeota (2.3% ± 3.5%), Cyanobacteria (2.3 ± 2.0%), Actinobacteria (1.9% ± 1.0%), and Acidobacteria (1.4% ± 1.0%) were also observed (Figure 3A). At the family level, Enterobacteriaceae (22.8% ± 20.0%), Lachnospiraceae (9.5% ± 8.8%), Ruminococcaceae (8.7% ± 4.2%), and Spiroplasmataceae (7.4% ± 17.5%) were predominant (Figure 3B).

The characteristics of the microbial composition in different organs of the four Tabanidae species were examined to analyze the variation in microbiota in tabanids. Firmicutes, Proteobacteria, and Bacteroidetes were the most dominant bacterial phyla in the various organs (Figure 3C). Notably, a high abundance of Tenericutes (20.9% ± 24.9%) was observed in the midguts of most tabanids. However, *Haematopota turkestanica* exhibited a lower abundance of Tenericutes (1.2% ± 0.7%) in its midgut than other tabanids. In Malpighian tubules, *Tabanus griseinus* and *Haematopota turkestanica* displayed higher abundances of Epsilonbacteraeota(5.0 ± 5.6 and 5.6% ± 7.5%). Furthermore, *Tabanus griseinus* exhibited a higher abundance of Tenericutes (11.3% ± 14.8%) and a lower abundance of Proteobacteria (16.8% ± 10.1%) in the Malpighian tubule. However, the overall structure of ovarian microbiota across the four tabanid species remained relatively consistent.

At the family level, Enterobacteriaceae (19.8% ± 22.9%), Lachnospiraceae (10.9% ± 11.1%), and Ruminococcaceae (9.7% ± 4.3%) emerged as the dominant families across the organs (Figure 3D). Notably, the abundance of Spiroplasmataceae (11.1% ± 14.8%) in the Malpighian tubules of *Tabanus griseinus* surpassed that in other tabanids. Additionally, higher levels of Enterobacteriaceae (23.7% ± 14.3%) and lower levels of Lachnospiraceae (7.1% ± 1.9) were observed in the Malpighian tubules of *Atylotus* sp. Spiroplasmataceae (29.1 ± 21.1 and 41.3% ± 38.3%) was notably abundant in the midgut of *Tabanus griseinus* and *Hybomitra* sp. Interestingly, the midgut of *Haematopota turkestanica* contains more Lachnospiraceae (6.7% ± 4.1%) but almost no Spiroplasmataceae (0.6% ± 0.6). It is noteworthy that compared to other tabanids, *Atylotus* sp. exhibited a higher enrichment of Enterobacteriaceae (33.2 ± 26.8 and 39.0% ± 15.9%) in both the midgut and ovary. The structures of the ovary microbiota of *Tabanus griseinus*, *Haematopota turkestanica,* and *Hybomitra* sp. were consistent, suggesting that the composition of the ovary microbiota may not be significantly influenced by genetic background.

**Figure 4:**
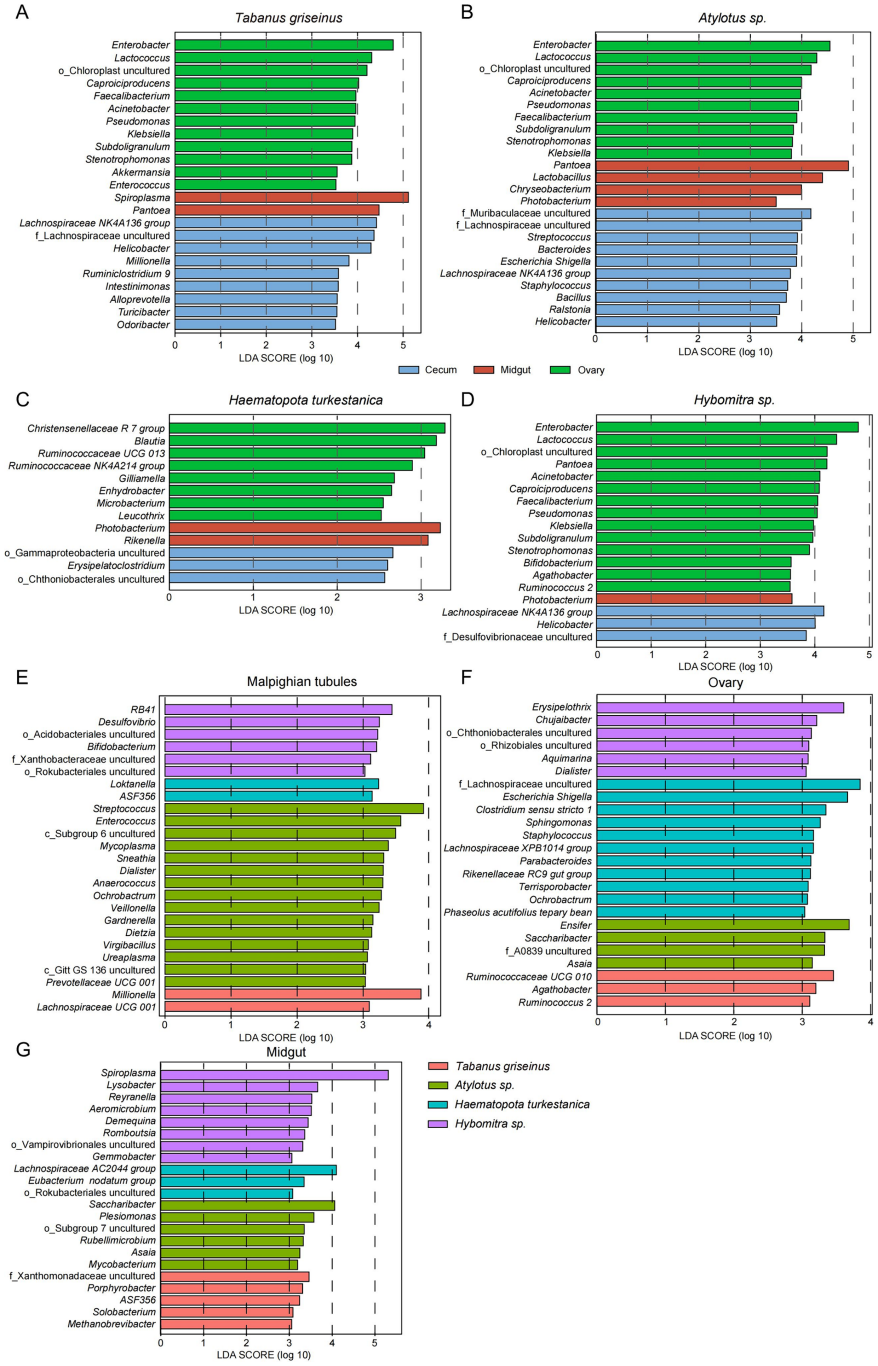
LEfSe analysis revealed differences in microbiota characteristics of the tabanids. The characteristic bacteria (Genus level) of organs of *Tabanus griseinus* **(A)**, *Atylotus* sp. **(B)**, *Haematopota turkestanica* **(C)**, and *Hybomitra* sp. **(D)** were analyzed using LEfSe. Then the bacteria characteristics (genus level) of different *Tabanus* were compared in Malpighian tubules **(E)**, ovary **(F)**, and midgut **(G)** respectively.

The main changes observed at the family level extended downstream to the hierarchical groups; therefore, we further examined the characteristics at the genus level. The relative abundances of dominant bacterial genera were not evenly distributed among each group (Supplementary Table S1), therefore, LEfSe analysis was used to detect characteristic genera in each group. This study confirmed consistent enrichment of *Lachnospiraceae* NK4A136 group and *Helicobacter* (LDA > 3, *p* < 0.05) in the Malpighian tubules of *Tabanus griseinus*, *Atylotus* sp., and *Hybomitra* sp. (Figures 4A,B,D). Notably, there was minimal difference in the microbiota of each organ in *Haematopota turkestanica*, with only three enriched genera (Figure 4C) found in the Malpighian tubules after lowering the threshold of the LDA score (LDA > 2, *p* < 0.05). A few bacteria were enriched in the midgut; however, *Photobacterium* was uniformly enriched in *Atylotus* spp., *Haematopota turkestanica,* and *Hybomitra* spp. In addition, only *Photobacterium* was significantly enriched in the midguts of *Hybomitra* spp. (Figure 4D).

The ovaries were enriched with the most characteristic bacteria, and similar to the Malpighian tubules, *Tabanus griseinus*, *Atylotus* spp., and *Hybomitra* spp. showed significant consistency (Figures 4A,B,D). *Enterobacter, Lactococcus*, *Caproiciproducens*, *Faecalibacterium*, *Acinetobacter*, *Pseudomonas*, *Klebsiella*, *Subdoligranulum*, and *Stenotrophomonas* were enriched in the ovaries (LDA > 3, *p* < 0.05). Interestingly, the ovarian microbiota of *Haematopota turkestanica* differed from that of the other tabanids, with bacteria such as *Christensenellaceae* R 7 Group, *Blautia*, and *Ruminococcaceae* UCG 013 being significantly enriched in the ovaries (Figure 4C).

LEfSe was employed to analyze how the microbiota varied across the organs of different species, revealing bacterial composition differences in the Malpighian tubules, midgut, and ovaries of each tabanid species. In the Malpighian tubules, *Atylotus* sp. was the most prominent genus (Figure 4E), with *Streptococcus* displaying the highest LDA value and was identified as the most significantly enriched genus (LDA > 3, *p* < 0.05). Furthermore, the genera most significantly enriched in *Tabanus griseinus*, *Haematopota turkestanica,* and *Hybomitra* sp. were *Millionella*, *Loktanella,* and RB41 (LDA >3, *p* < 0.05). In the midgut samples collected from the Hybomitra sp., yet in the context of an expected microbiome diversity, we observed *Spiroplasma* being the most significantly enriched (Figure 4G). The uncultured F_Xanthomonadaceae, *Saccharibacter,* and *Lachnospiraceae* AC2044 *group* were the most significantly enriched genera in *Tabanus griseinus*, *Atylotus* sp., and *Haematopota turkestanica,* respectively.

Consistent with the organ-level analysis, *Haematopota turkestanica* exhibited the most specialized ovarian microbiota, thus having the highest number of marked genera (Figure 4F). F_Lachnospiraceae uncultured and *Escherichia/Shigella* were the most abundant bacteria in the ovaries. The most significantly enriched genera in the ovaries of *Tabanus griseinus*, *Atylotus* spp. and *Hybomitra* spp. were *Ruminococcaceae* UCG 010, *Ensifer*, and *Erysipelothrix*, respectively.

### 3.5 Putative pathogens in the tabanids

**Figure 5:**
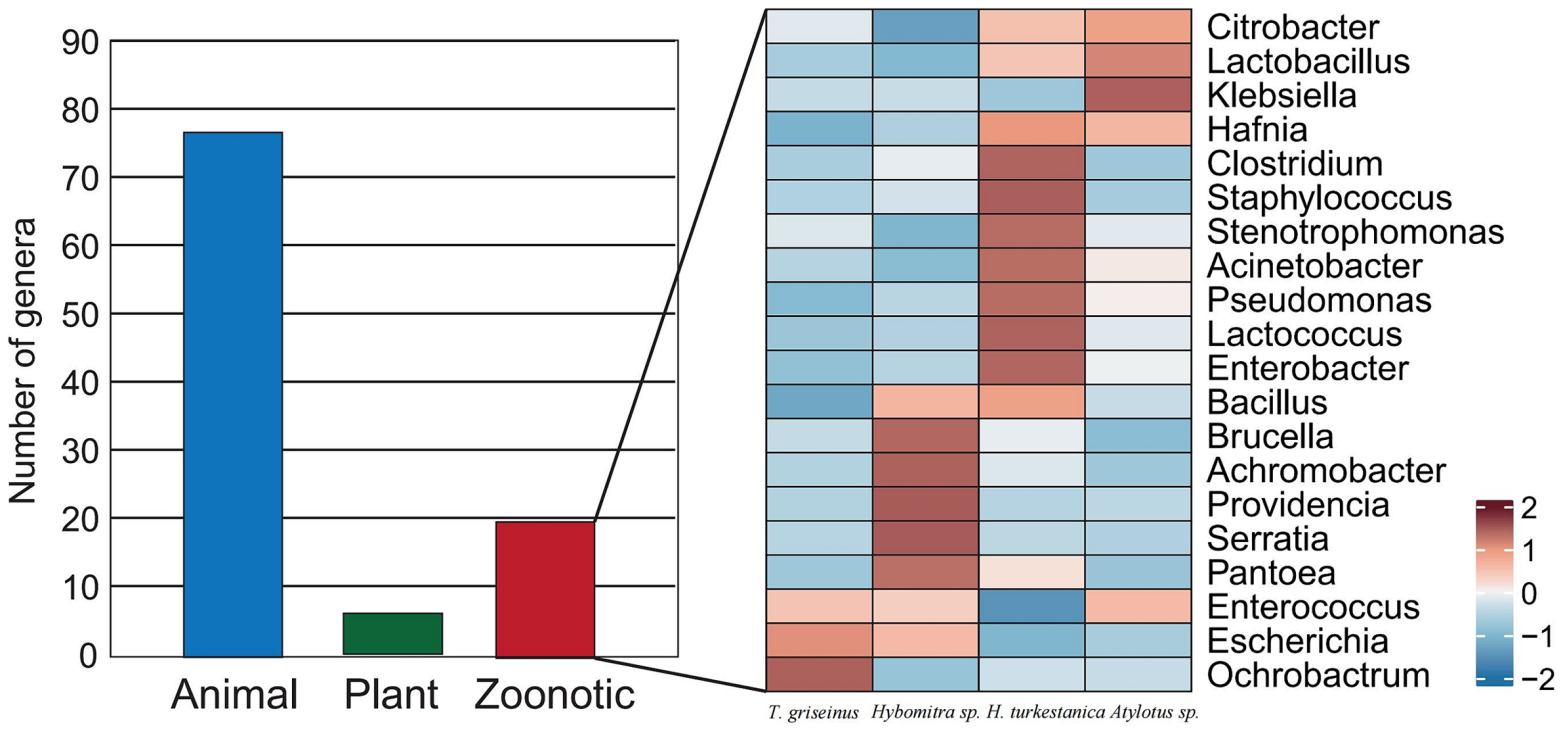
The composition of putative pathogens and the microbial index of pathogenic bacteria in the tabanid microbiota.

Putative pathogens within the microbiota of tabanids were identified using a pathogen detection pipeline to assess the potential public health risks associated with tabanids. The MBPD is known for its ability to detect a broad spectrum of animal, plant, and zoonotic pathogens. The results revealed over 100 genera of putative animal pathogens were the most abundant (Figure 5). Among these, 20 genera were annotated as zoonotic agents, with *Citrobacter* exhibiting the highest abundance among the zoonotic pathogens. Interestingly, *Brucella* species had varying abundance distributions among the four fly species, highlighting the presence of this important group of zoonotic pathogens (Figure 5).

### 3.6 Microbiota impacting on vectors

Research on vector-microbiota interactions has revealed the significant impact that microbiota can have on various biological traits in disease-transmitting vectors. This change in the composition of the vector microbiota can potentially influence pathogenic infections through diverse mechanisms. Microbiota may directly interact with vector pathogens or modulate pathogen infection by regulating host immune defenses and nutritional status. The influence of the microbiota on other aspects of vector physiology and pathogen transmission, as observed in the microbiome of tabanids, is summarized in Table 2.

**Table 2:** Microbiota and potential biological functions in various *Tabanus*.

Microbiota	Impacts on vectors	Species
*Acinetobacter* spp.	Promote food digestion	*Tabanus griseinus, Atylotus* sp., *Haematopota turkestanica*, and *Hybomitra* sp.
*Serratia* spp.	Inhibit Plasmodium infection	*Tabanus griseinus*, *Atylotus* sp., *Haematopota turkestanica*, and *Hybomitra* sp.
*Rahnella* spp.	Facilitate Leishmania infection	*Haematopota turkestanica* and *Atylotus* sp.
*Rhodococcus* spp.	Inhibit trypanosome infection	*Haematopota turkestanica* and *Atylotus* sp.
*Klebsiella* spp.	Attracts oviposition	*Tabanus griseinus*, *Atylotus* sp., *Haematopota turkestanica*, and *Hybomitra* sp.
*Chryseobacterium* spp.	Fecundity	*Tabanus griseinus*, *Atylotus* sp., *Haematopota turkestanica*, and *Hybomitra* sp.
*Asaia* spp.	Activates antimicrobial peptides	*Tabanus griseinus* and *Atylotus* sp.
*Spiroplasma* spp.	Protect host from parasitic wasps and nematodes	*Tabanus griseinus*, *Atylotus* sp., *Haematopota turkestanica*, and *Hybomitra* sp.

## 4 Discussion

Interest in microbial communities associated with disease vectors has grown in recent years. This interest stems from understanding that interactions between bacteria can influence the survival and transmission of pathogens (Song et al., 2022; Laukaitis and Macaluso, 2021; Wu et al., 2019). Despite this increased focus, many arthropod-associated human diseases remain undiagnosed, and knowledge of the prevalence, diversity, and pathogenicity of novel arthropod-borne pathogens is limited. The necessity for ongoing microbiological surveillance of vectors is evident. Differences in microbiota composition have been extensively documented in other vector arthropods, including variations in vector species, sex, organs, and developmental stages (Strand, 2018; Muturi et al., 2017; Choubdar et al., 2021). This study focused on the differences in the microbiota among tabanids, considering two factors: organs and species. The research observed that diversity of the microbiota in the ovaries was greater than that in other organs across all tabanids. However, differences in microbiota diversity among different tabanid species were only evident in the ovarian samples of *Atylotus* spp., suggesting that microbiota diversity in different organs may be independent.

Interestingly, previous studies have found that the microbiota diversity in the ovary is higher than that in the digestive tract (Malpighian tubules and midgut), which contrasts with observations in hard ticks (Guizzo et al., 2020). This reduction in diversity may be associated with blood feeding (Qiu et al., 2022). Studies have also revealed that the gut bacterial diversity of *Aedes aegypti* decreases after feeding on blood (Muturi et al., 2019), although it returns to its original pre-blood meal levels as the blood meal is digested. Considering that wild tabanids typically feed on various hosts and conducting artificial feeding experiments under laboratory conditions presents challenges, it is important to acknowledge the potential influence of blood meal sources on microbiota dynamics. Hence, additional research is necessary to clarify the effect of blood meals on the microbiota of tabanids. The midgut is the primary site for bacterial infection, colonization, and subsequent spread among BFA, with the relative abundance of these bacteria varying from one host to another. The microbiota found in the tabanids exhibited a core composition of Firmicutes and Proteobacteria at the phylum level. Tenericutes were the dominant phyla in *Tabanus griseinus* and *Hybomitra* sp., whereas Proteobacteria were the dominant phyla in *Atylotus* sp. and *Haematopota turkestanica*. Tenericutes are frequently found in insects such as spiders and bees (Magagnoli et al., 2022). Most Tenericute species in the midgut of *Tabanus griseinus* and *Hybomitra* spp. belong to Spiroplasmataceae, which were recently reclassified under the phylum Mycoplasmatota (Oren and Garrity, 2021). This bacterium is widespread in various arthropods and has been extensively described in ticks (Binetruy et al., 2019). Spiroplasmataceae are transmitted between arthropods through maternal inheritance and horizontal transfer (Bell-Sakyi et al., 2015). Enterobacteriaceae, a representative family of Proteobacteria in *Atylotus* sp. and *Haematopota turkestanica*, has been reported in *A. aegypti* mosquitoes (Macleod et al., 2021; David et al., 2016) and is considered a potential pathogen. Members of this family are closely associated with viral infection and transmission (Wu et al., 2019; Kozlova et al., 2021). The representative family of Firmicutes is Lachnospiraceae, which has also been reported in *A. aegypti* (Arévalo-Cortés et al., 2022) and is associated with mucin degradation in the gut (Vacca et al., 2020).

A single food source, such as blood, causes the arthropod host to be deficient in essential metabolites, such as thiamine, pyridoxine, folic acid, and other B vitamins (Rio et al., 2016). These metabolites are crucial for arthropods, and ectoparasites rely on their microbiota to compensate for the missing nutrients. Unlike non-blood-feeding insects, certain gut bacteria in blood-sucking arthropods benefit their hosts by upregulating numerous genes that encode essential nutrients (Sonenshine and Stewart, 2021). Different blood-sucking arthropods harbor diverse composition of their microbial communities that provide essential nutrients to the host during the long-term adaptation process to compensate for genome loss (Duron and Gottlieb, 2020). Three phyla (Firmicutes, Actinomycetes, and Proteobacteria) and eight bacterial families (Bacteriaceae, Rickettsiaceae, Anaplasmodiaceae, Enterobacteriaceae, Sphingomonas, Moraxellaceae, Pseudomonas, and Staphylococcaceae) were identified in most blood-sucking arthropods (Clow et al., 2018). Ruminococcaceae are abundant in the cecum and midgut of tabanids, and members of this family have been reported in both hard ticks (Xu et al., 2023) and black soldier flies (Wu et al., 2022). This family comprises an important class of organolytic bacteria that aid in the digestion of host and metabolism nutrients (Alvarado et al., 2021).

Our study revealed a relatively consistent microbiome in the ovaries of various tabanid species, suggesting the conservation of vertically transmitted bacteria across the tabanid family. The predominant bacterial families identified in ovaries were Rumenococcaceae, Enterobacteriaceae, Trichomyceaceae, Lactobacillaceae, and Streptococcaceae. The microbiota present in the ovaries may play a role in facilitating viral infection of arthropods, as observed in mosquitoes (Díaz et al., 2021; Caragata et al., 2021). Further studies examining viral carriage in horsefly ovaries are necessary to elucidate the relationship between these bacteria and pathogens.

The midgut and Malpighian tubules of BFA serve as primary sites of viral invasion (Wu et al., 2019). The intricate community of symbiotic microbiota in these organs can regulate host defense against viral invasion or entry into the intestinal epithelium. For instance, *Wolbachia*, a symbiotic bacterium in mosquitoes, interferes with mosquito-borne viral replication, likely through alterations in immune or physiological responses (Shi et al., 2023; Hajdušek et al., 2013). Similarly, *Serratia marcescens*, a bacterium known to promote dengue virus infection in mosquitoes (Wu et al., 2019) was identified in the midgut samples of tabanids. Therefore, targeting midgut microbiota is a promising approach for interventions to control and prevent insect-borne diseases.

*Spiroplasma* is a bacterium widely distributed in various tissues such as the hemolymph, adipose bodies, and salivary glands of ticks, and can be transmitted vertically through the ovaries (Ballinger and Perlman, 2017). Previous studies have demonstrated that *Spiroplasma* can confer protection against parasitic wasps and nematodes (Wang et al., 2023). However, the role of *Spiroplasma* in susceptibility to arbovirus vectors remains poorly understood. Our findings indicate that *Spiroplasma* is highly abundant in the ovaries of tabanids, suggesting that tabanids may harbor a vertically transmitted protective microbiota similar to that observed in other arthropods.

The presence of pathogenic bacteria in blood-sucking arthropods poses a significant public health risk, and is a key area of current research (Van Treuren et al., 2015). However, tabanids have received relatively little attention compared to other hematophagous Diptera species. The MBPD results highlight the potential risks associated with tabanids. For instance, *Brucella* is an important pathogen that affects both humans and animals, and can cause abortion in animals, leading to economic losses and impacting animal health (Suárez-Esquivel et al., 2020). Although cases transmitted by horseflies are rarely reported, the possibility that these flies serve as neglected vectors for transmission cannot be disregarded, whereas other detected pathogens rarely infect healthy populations. However, infections caused by opportunistic pathogens can complicate diagnosis and treatment and pose a serious threat to immunocompromised patients owing to their multidrug resistance (Sheppard, 2022). Therefore, despite conducting an in-depth analysis of potentially pathogenic bacteria in the tabanid microbiota, further research is warranted to obtain a comprehensive understanding of the public health risks associated with pathogenic microorganisms carried by the microbiota of tabanids.

The microbiota of BFA can be influenced by a variety of biological and abiotic factors at both the macro (population) and micro (organ/tissue) levels. Geography is a significant factor that influences variation in the bacterial communities of BFA (Thapa et al., 2019; Yu et al., 2022). It is influenced by factors such as host activity, temperature, humidity, and sunlight exposure. This suggests the potential for tracing the origin of quarantined insects using microbial community characteristics. The sampling site for this study was Daqing City, Heilongjiang Province, China, known for its abundant wetland resources supporting various tabanid species. However, the limited scope of our sampling site may constrain the generalizability of our findings, highlighting the need for a broader sample collection to comprehensively understand the symbiotic microbiota of tabanids.

## 5 Conclusion

This study comprehensively analyzed the microbiota composition of four common tabanid species. Significant variations in microbial community characteristics were observed across various organs, with notably high diversity detected in the ovaries. The similarities in bacterial taxa among the ovaries of the four tabanid species suggest potential conservation of vertically transmitted bacteria in these BFA. Moreover, distinct microbiota characteristics in the digestive tracts of the four tabanid species may be closely linked to their specific ecological niches and behaviors. Future studies should explore the intricate relationships among tabanid microbiota, habits, and habitats, as well as their potential roles as vectors of pathogens. These insights could aid in the geographical tracking of parasite species and the development of symbiotic-based strategies for controlling insect-borne diseases.

## Data Availability

The original contributions presented in the study are publicly available. This data can be found here: https://www.ncbi.nlm.nih.gov/bioproject/PRJNA1119251/.
